# Impact of Viscosity on Human Hepatoma Spheroids in Soft Core–Shell Microcapsules

**DOI:** 10.1002/adhm.202302609

**Published:** 2024-01-25

**Authors:** Xuan Peng, Željko Janićijević, Sandy Lemm, Sandra Hauser, Michael Knobel, Jens Pietzsch, Michael Bachmann, Larysa Baraban

**Affiliations:** ^1^ Helmholtz‐Zentrum Dresden‐Rossendorf Institute of Radiopharmaceutical Cancer Research 01328 Dresden Germany; ^2^ Faculty of Medicine and University Hospital Carl Gustav Carus Technische Universität Dresden 01307 Dresden Germany; ^3^ Faculty of Chemistry and Food Chemistry School of Sciences Technische Universität Dresden 01062 Dresden Germany; ^4^ Helmholtz‐Zentrum Dresden‐Rossendorf Institute of Fluid Dynamics 01328 Dresden Germany; ^5^ National Center for Tumor Diseases (NCT) 01307 Dresden Germany; ^6^ German Cancer Research Center (DKFZ) 69120 Heidelberg Germany; ^7^ German Cancer Consortium (DKTK) 01307 Dresden Germany

**Keywords:** cell adaptation, cytoskeleton, hepatoma spheroids, hydrogel microcapsules, tumor microenvironment

## Abstract

The extracellular environment regulates the structures and functions of cells, from the molecular to the tissue level. However, the underlying mechanisms influencing the organization and adaptation of cancer in three‐dimensional (3D) environments are not yet fully understood. In this study, the influence of the viscosity of the environment is investigated on the mechanical adaptability of human hepatoma cell (HepG2) spheroids in vitro, using 3D microcapsule reactors formed with droplet‐based microfluidics. To mimic the environment with different mechanical properties, HepG2 cells are encapsulated in alginate core–shell reservoirs (i.e., microcapsules) with different core viscosities tuned by incorporating carboxymethylcellulose. The significant changes in cell and spheroid distribution, proliferation, and cytoskeleton are observed and quantified. Importantly, changes in the expression and distribution of F‐actin and keratin 8 indicate the relation between spheroid stiffness and viscosity of the surrounding medium. The increase of F‐actin levels in the viscous medium can indicate an enhanced ability of tumor cells to traverse dense tissue. These results demonstrate the ability of cancer cells to dynamically adapt to the changes in extracellular viscosity, which is an important physical cue regulating tumor development, and thus of relevance in cancer biology.

## Graphical abstract

Cancer cells have a remarkable capacity to dynamically adapt to fluctuations in extracellular viscosity. Here HepG2 human hepatoma cell spheroids are cultivated within 3D microcapsule reactors created using the high‐throughput droplet‐based microfluidics. The image showcases alterations in the expression and distribution of F‐actin and keratin 8 in response to varying viscosities of the surrounding medium within the microcapsules.

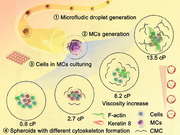

## 1 Introduction

Cell proliferation and tissue development are influenced by the mechanisms of cellular response to various physical stimuli, such as fluid shear stress,^[^ ^1^ ^]^ stiffness,^[^ ^2^ ^]^ and matrix stress.^[^ ^3^ ^]^ The tremendous interest of the community in investigating the effects of physical stimuli on cell constructs has led to the understanding that cell behavior is directly affected by the mechanical properties of the culturing environment. For example, cells cultured in a high‐viscosity medium exhibit decreased cell length and reduced cell mobility.^[^ ^4^ ^]^ When upscaling to the microtissue level (described in Table S1 in the Supporting Information), viscoelasticity of the matrix tends to play an important role, inducing the symmetry breaking of spheroids and leading to the formation of invasive finger‐like protrusions.^[^ ^2^ ^]^ Cells and their extracellular environment form a unique ecosystem, by mutually influencing each other: extracellular space changes upon differentiation of physiological and pathological states, and the other way around, the environmental changes influence cell metabolomics, as well as, cellular sensing and response mechanisms.^[^ ^5^ ^]^

Compared to normal healthy cells, malignant tumor cells demonstrate increased adaptability of their mechanical properties when exposed to mechanical stimuli.^[^ ^6^ ^]^ Their ability to sense the environment and adapt accordingly enhances the metastatic potential of tumor cells. For instance, solid tumors, such as breast or cervical ones are often more rigid than the surrounding healthy tissues.^[^ ^7^, ^8^ ^]^ However, the rigid tumor cores may also be encapsulated by softer “layers” of cells that are more deformable, in comparison with those in healthy tissue and rigid cores. This decreased stiffness potentially enhances the ability of the tumor to navigate through the surrounding tissues.^[^ ^8^, ^9^ ^]^ While there is a growing interest in investigating the influence of mechanical stimuli on cellular properties (see details in Table S2 in the Supporting Information) among scientists from various disciplines, our current understanding of cancer cell clustering in response to physical, and particularly mechanical properties of the environment, remains limited. Therefore, further investigations are necessary to expand our knowledge in this area.

adaptation of cancer in three‐dimensional (3D) multicellular organoids and spheroids have emerged as crucial model tools for cancer research, bridging the gap between 2D monolayer models and in vivo solid tumors.^[^ ^10^, ^11^, ^12^ ^]^ Tumor spheroids can be generated in scaffold‐free conditions or with the incorporation of 3D scaffolds.^[^ ^13^, ^14^, ^15^ ^]^ Scaffold‐free methods include liquid overlay,^[^ ^16^ ^]^ hanging drop,^[^ ^17^ ^]^ and microfluidic device‐based technology.^[^ ^18^ ^]^ 3D scaffolds are constructed using various biomaterials ranging from natural biopolymer compositions (collagen,^[^ ^19^ ^]^ alginate,^[^ ^20^ ^]^ and matrigel^[^ ^21^ ^]^), to synthetic polymers, such as chitosan,^[^ ^22^ ^]^ polycaprolactone,^[^ ^23^ ^]^ and poly(ethylene glycol) diacrylate.^[^ ^24^ ^]^ Droplet‐based microfluidic technology, which opens remarkable opportunities for miniaturization and parallelization, has recently emerged as a versatile tool in bio(chemical) reactions and analysis. This technology provides large‐scale quantitative 3D extracellular environments for high‐throughput analysis, including liquid droplets,^[^ ^25^, ^26^ ^]^ gel beads,^[^ ^27^, ^28^ ^]^ and capsules.^[^ ^29^, ^30^ ^]^ In addition, the droplet‐based microfluidic approach enables fabrication suitable for engineering cell spheroids and organoids.^[^ ^30^, ^31^, ^32^, ^33^ ^]^ In our previous study,^[^ ^29^ ^]^ we utilized this approach to optimize microcapsule generation and investigate the differences in proliferation and aggregation behaviors of HepG2 cells caused by the changes in capsule shell properties (thickness, chemical composition, and gelation time) which affect shell permeability. Furthermore, a method utilizing cancer spheroids has been employed to study the immunological behavior of antigen‐specific T cells in vitro.^[^ ^26^ ^]^ To date, most of these in vitro cell functional assays in beads or capsules have been conducted either in a medium with viscosities similar to that of water (0.7 cP at 37 °C) or within a soft gel matrix.^[^ ^34^, ^35^ ^]^ However, the viscosities of extracellular fluids, including interstitial fluid, blood plasma, lymph, and transcellular fluid change under different physiological conditions.^[^ ^4^, ^5^ ^]^ For example, the viscosity of interstitial fluid is close to 3.5 cP, and that of plasma is ≈2 cP.^[^ ^1^, ^36^ ^]^ Interestingly, primary tumor development is characterized by the increase of microenvironment viscosity due to the accumulation of macromolecules over time.^[^ ^5^ ^]^ The viscosity of whole blood is typically <6 cP, but it can exceed 8 cP in case of pathological abnormalities.^[^ ^5^, ^37^ ^]^ Thus, it is confirmed that the mechanical properties of the extracellular environment make a significant impact at the single‐cell level.^[^ ^5^ ^]^ However, we still need to advance our understanding of the response of larger cell assemblies (such as spheroids) to alterations of microenvironment viscosity in order to gain insights into fundamental mechanisms and develop more accurate in vitro cancer models.

**Figure 1:**
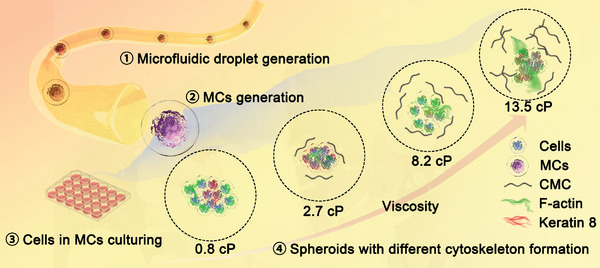
Illustration of microcapsule generation and HepG2 cell spheroid adaptation under different viscosity conditions. (MCs: microcapsules; CMC: carboxymethylcellulose). HepG2 cells were cultured in microenvironments with varying viscosities. When cultured in a standard cell culture medium lacking any physical matrix support, the cells exhibited a tendency to aggregate into clusters, accompanied by increased expression of keratin 8. This aggregation resulted in the formation of a solid core, essential for cell attachment and subsequent proliferation. With an increase in viscosity to 2.7 cP, the cells displayed a reduced propensity to aggregate, leading to the formation of smaller spheroids compared to the control (0.8 cP). Subsequently, at higher viscosities of 8.2 cP or beyond, cells on the spheroid periphery exhibited enhanced expression of F‐actin and developed pseudopodia, indicative of a softer cellular phenotype and increased migratory potential.^[^ ^42^, ^43^ ^].^

In this study, we focused on spheroids derived from HepG2 cells, which are used both as a model for liver function and as a model for hepatocellular carcinoma,^[^ ^38^, ^39^ ^]^ to investigate the influence of fluid viscosity on 3D cell assemblies (**Figure** **1**). To perform the investigation, we generated core–shell microcapsules (MCs) with diameters of ≈500 µm as bioreactors for cell culturing using the microfluidic platform that we designed previously.^[^ ^29^ ^]^ The shell of the MCs is composed of a soft alginate (AL), while the core of the MCs contains a cell culture medium with adjusted viscosity. The viscosity of the core liquid is modulated by incorporating a predetermined amount of carboxymethylcellulose (CMC). Specifically, AL‐CMC MCs with core liquid viscosities of 0.8, 2.7, 8.2, and 13.5 cP were designed using a droplet‐based approach. CMC macromolecule with no natural binding sites was selected to avoid cell‐matrix interactions so that cells can freely probe their extracellular surroundings.^[^ ^40^, ^41^ ^]^ As a control, HepG2 cells were also cultured within the soft 3D alginate gel beads (AL GBs) generated under the same conditions. Cell proliferation, spheroid growth, and morphological changes were compared between MCs and GBs. Immunostaining was employed to examine the organization of the cytoskeleton in different viscous environments. This research illustrates the difference in HepG2 cell proliferation, aggregation, and cytoskeleton distribution resulting from the response to viscosity changes within a 3D environment. We believe that this study not only introduces a method for the design and engineering of cancer spheroids and organoids but also offers valuable insights into the regulation of tumor assembly development by the mechanical properties of microenvironments, which is of high relevance for cancer biology.

## 2 Results and Discussion

### 2.1 Microcapsules with Different Core Viscosities

**Figure 2:**
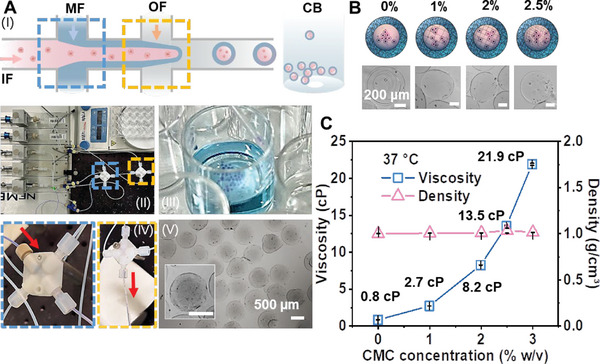
AL & AL‐CMC microcapsules (MCs). A) (I&II) Schematic diagram and photos of the droplet microfluidic system to fabricate AL MCs and AL‐CMC MCs (IF: inner fluid, cell culture medium with/without CMC; MF: middle fluid, sodium alginate (NaAL) with sodium dodecyl sulfate (SDS); OF: outer fluid, hexane; CB: CaCl_2_ bath; 1 mg mL^−1^ toluidine blue solution was used to dye the capsules in (III)); (III) MC collection wells; (IV) Cross junctions for co‐flow fluid (left) and droplet generation (right); (V) Representative images of MCs (scale bar = 500 µm). B) Schematic diagram of cell aggregation and representative images of AL MCs and AL‐CMC MCs. Scale bar = 200 µm. C) Viscosity and density of DMEM containing different concentrations of CMC (0, 1, 2, 2.5, and 3% w/v) at 37 °C.

To study the impact of extracellular fluid viscosity on the proliferation behavior of HepG2 cells in a 3D environment, we designed the MCs by incorporating CMC into the cell medium. Initially, we generated the MCs (diameter ≈570±100 µm; shell thickness ≈85±20 µm) containing the cell culture medium with CMC and cells in the core at room temperature (20 °C). MCs were subsequently incubated at 37 °C. The setup for capsule generation and cross junction system are depicted in **Figure** **2**AI–IV, while the core–shell structure of AL MCs is clearly shown in Figure 2BV.

To determine the viscosity of the MC core liquids, we measured the viscosities of core liquids containing Dulbecco's Modified Eagle Medium (DMEM) and different concentrations of incorporated CMC using a viscometer (see details in Section 4.2.), as depicted in Figure 2C. The viscosity of the cell culture medium with different concentrations of CMC (from 0 to 3% w/v) ranges from 0.8 to 21.9 cP at 37 °C. At the same time, the cell culture medium density remains relatively constant across different temperatures and CMC concentrations. For details on the viscosity measurement refer to Supporting Information (Figure S1 and Table S3, Supporting Information).

For subsequent investigations, we selected representative capsules with CMC concentrations of 0, 1, 2, and 2.5% w/v, referred to as AL MCs, AL‐CMC1 MCs, AL‐CMC2 MCs, and AL‐CMC2.5 MCs, respectively. These capsules provided low (0.8 cP), intermediate (2.7 cP), moderately high (8.2 cP), and high viscosity (13.5 cP) environments in the core, allowing us to study the effect of viscosity on cell behavior. Since the proliferation of cells inside gel beads differs from the proliferation inside capsules, we used GBs (diameter ≈690±200 µm; generated using the same flow rates as for MCs) as a control.

### 2.2 Diffusion of Sodium Fluorescein into AL MCs, AL‐CMC MCs, and AL GBs

**Figure 3:**
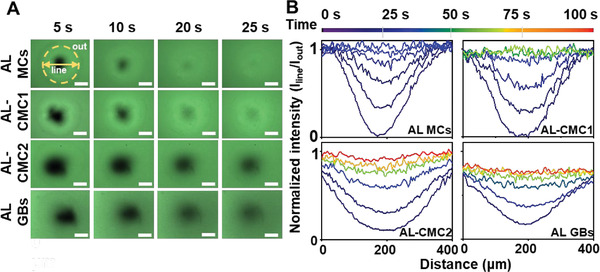
Diffusion of sodium fluorescein (FSC, 376 Da) into AL MCs, AL‐CMC MCs & AL GBs. A) Representative fluorescent micrographs obtained after immersion in a 5 × 10^−3^ m solution of FSC (scale bar = 200 µm). B) Midline/outer fluorescence intensity ratio for MCs with 0%, 1%, and 2% of CMC (AL MCs, AL‐CMC1, and AL‐CMC2), and AL GBs at different time points.

Before the culturing of cells in MCs, we confirmed that the capsule structure allows for efficient mass transfer of nutrients and metabolites through the shell.^[^ ^29^ ^]^ Higher viscosity of the fluid is expected to reduce the diffusion rate of the molecules. To assess the impact of increased viscosity on the rate of nutrient exchange (e.g., glucose, molecular mass (*M*_r_) of 180 Da and amino acids, *M*_r_ = 80–210 Da) within the MCs during a specific period, we conducted a diffusion experiment using similarly sized model molecule – sodium fluorescein (FSC, *M*_r_ = 376.27 Da, 5 × 10^−3^ m solution), and visualized FSC permeation using a confocal microscope. We acquired fluorescent micrographs at different time points from 0 to 120 s for MCs and GBs (*n* ≥ 3) and then extracted the corresponding intensity profiles from the midline profile. **Figure** **3**A,B demonstrates that the increase in viscosity inside the capsule core leads to a decrease in the diffusion rate of FSC during the loading of MCs. The equilibrium between the core and the external medium is reached after ≈30 s for AL MCs and 50 s for AL‐CMC1 MCs. The diffusion of FSC in AL‐CMC2 MCs follows a similar trend as in the AL GBs, taking about 100 s to reach equilibrium. The diffusion rate of FSC is the lowest in AL‐CMC2.5 MCs, requiring ≈120 s to reach equilibrium (Figure S2, Supporting Information).

### 2.3 Proliferation of Cells and Formation of Spheroids in MCs and GBs

**Figure 4:**
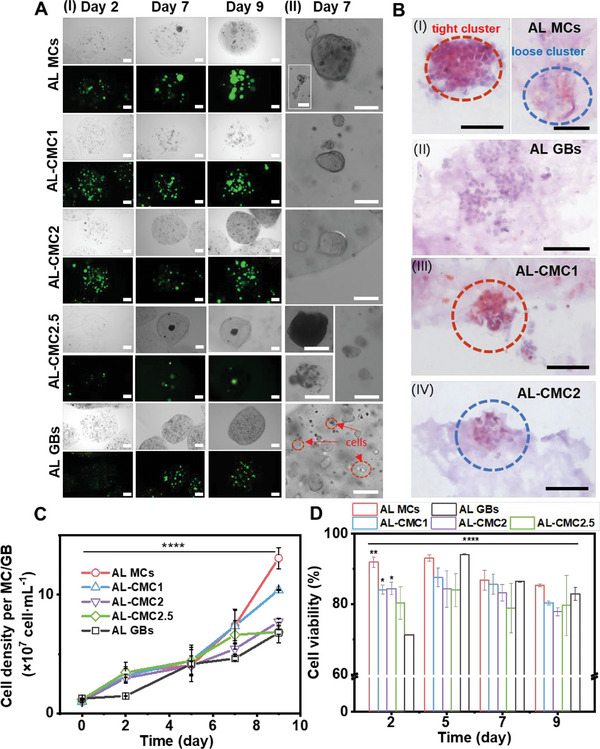
Cells and spheroids in AL MCs, AL‐CMC MCs & AL GBs. A)  Representative optical and live/dead (green/red) fluorescent staining images of cell and spheroid proliferation in AL MCs, AL‐CMC MCs & AL GBs loaded with liver tumor spheroids (I: scale bar = 200 µm; II: scale bar = 100 µm). B) Representative hematoxylin and eosin B (H&E) staining of cell clusters in MCs & GBs (scale bar = 100 µm). C) Average cell density obtained for each MC or GB. Average cell density in AL GBs is considered as a control. Data are reported as mean ± standard deviation (SD). *p*‐values were calculated using the two‐way ANOVA test combined with Tukey's multiple comparison test, ****p < 0.0001, *n* ≥ 3. D) Cell viability trends in MCs compared with GBs. Data are reported as mean ± standard deviation (SD). *p*‐values were calculated using the two‐way ANOVA test combined with Dunnett's multiple comparison test, *p < 0.0332, **p < 0.0021, and ****p < 0.0001, *n* ≥ 3.

After verifying the MC permeability, we generated the MCs (with different concentrations of incorporated CMC in the core: 0%, 1%, 2%, and 2.5% w/v) and GBs containing HepG2 cells as models of hepatocellular carcinoma.^[^ ^38^, ^39^ ^]^ **Figure** **4** summarizes the findings about the character of assembly, proliferation, and viability of HepG2 cells and spheroids grown within different groups of MCs and GBs (used as control reactors).

**Figure 5:**
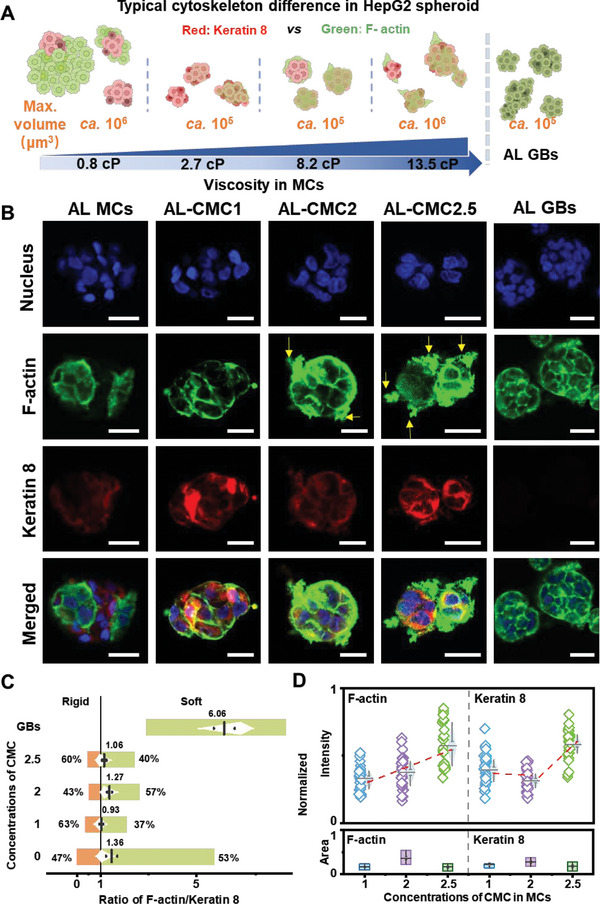
Cytoskeleton differences between spheroids in AL MCs & AL‐CMC MCs. A) Illustration of typical cytoskeleton and volume changes of spheroids related to medium viscosity. Red spheroids represent increased keratin 8 expression, green spheroids represent increased F‐actin expression, and the extension area of green represents F‐actin extrusion. B) Immunostaining of spheroids in MCs (nucleus: Hoechst, blue; F‐actin: phalloidin, green; keratin 8: cytokeratin 8, red; scale bar = 20 µm). C) Average intensity ratio of F‐actin and keratin 8 in spheroids in different MCs (*n* = 30) and GBs (*n* = 18). D) Average intensity and distribution area of F‐actin and keratin 8 in spheroids in different AL‐CMC MCs (*n* = 30).

Cells in MCs and GBs show radically distinct aggregation and proliferation trends. While the growth in GBs is characterized by more dispersed cell assemblies of smaller size (gel matrix does not support extensive cell migration but offers mechanical support), the cells tend to aggregate into one or several large clusters inside MCs. In such a manner, cells create mutual mechanical support, compensating for the absence of rigid interfaces inside the liquid MC core. Increased viscosity of liquid MC core may affect the character of collective cell behavior, which is described in Figures 4 and Figure 5. In accordance with our previous results,^[^ ^29^ ^]^ in this study we also observed large‐scale cell spheroids within MCs with 0% CMC in the core and highly dispersed small cell clusters within GBs (Figure 4A). Optical and fluorescent images recorded during the first 9 d of culturing (Figure 4A; Figure S3, Supporting Information) reveal that cells aggregate faster in pristine AL MCs than in AL‐CMC MCs with more viscous core or AL GBs. This is confirmed by counting the average number of spheroids per MC and GB (Figure S4, Supporting Information). The earlier the number of spheroids per capsule reached its highest value, the quicker the cells aggregated. With the increase in viscosity of the surrounding medium, spheroids tend to grow into smaller cell aggregates. Spheroids in AL GBs (Figure 4A) grew similarly as in AL‐CMC2.5 MCs (Figure S3, Supporting Information), indicating that many cells either did not aggregate or formed only small spheroids within 7 d. However, we observed that some HepG2 cells in AL‐CMC2.5 MCs aggregated into large spheroids comparable to those in AL MCs after 7 d. These large spheroids eventually formed tight clusters, but their viability decreased rapidly (within 2 d) due to nutrient deprivation.

To further quantify the impact of viscosity on cell aggregation, we analyzed 50 randomly selected spheroids from each group by calculating their volumes and circularities (Figures S5–S7, Supporting Information). Briefly, spheroids in AL MCs showed a trend of faster growth compared to the spheroids in AL‐CMC1 and AL‐CMC2 MCs over 9 days (Figure S5, Supporting Information). The average volume of spheroids in AL GBs was the lowest. Furthermore, there is a visible impact of viscosity on the shape of spheroids. Analysis of circularity shows that the viscous environment promotes cell aggregation into rounder assemblies (Figures S6 and S7, Supporting Information).

To compare the differences in cell proliferation, we calculated the average cell density per MC or GB and measured the viability of cells (Figure 4C,D, see details in Section 4.7.). There are no significant differences between MC groups at the early stage (5 d), but the cells in MCs proliferate faster compared to the cells in GBs (Figure 4C). After 5 d, the cells in AL‐CMC MCs show lower proliferation rates compared to the cells in AL MCs. The viability of cells in each group peaked on day 5 and then slowly declined, which can also be attributed to the lower cell proliferation rates due to the lack of nutrients (Figure 4D). Although the cells could still proliferate after 9 d in the AL‐CMC1 MCs and AL GBs (Figure S8, Supporting Information), many MCs were broken into fragments (Figure S9, Supporting Information). In AL‐CMC2 MCs and AL‐CMC2.5 MCs, most spheroids ceased to grow after 12 and 9 d, respectively, due to nutrient depletion.

Since cell proliferation behavior on a 2D plate is significantly different from the proliferation behavior in a 3D culture, 2D culturing experiments were also carried out as a control (Figure S10, Supporting Information). Cells cultured without CMC (0%) tended to aggregate into small islands. As the viscosity increased, cells were more inclined to proliferate by attaching to the plate surface. These results align with a recent study suggesting that increased extracellular fluid viscosity enhances cell migration on a 2D surface.^[^ ^5^ ^]^ We did not observe significant differences in cell viability for different concentrations of incorporated CMC (all values exceeded 80%, see Figure S11 in the Supporting Information).

### 2.4 Mechanical Adaptation of Spheroids to Different Medium Viscosities in MCs

Results presented above demonstrate that the increase of medium viscosity in the liquid core of the microcapsules leads to the decreased proliferation period as well as the changes in distribution, size, and shape of the spheroids. When analyzing this information together with previously described permeability results (Figure 4), we conclude that cancer cells placed in the capsules with highly viscous cores visually start to behave as in the completely gelled microbeads. While the macroscopic properties of cell assemblies (spheroids) have been elucidated, a key question that remains is whether the mechanical alterations in the surrounding environment directly influence the behavior of individual cells. Notably, the ability of tumor cells to dynamically adapt to the mechanical properties of the environment is currently one of the most crucial problems in understanding cancer.^[^ ^6^ ^]^

Histological analysis with hematoxylin and eosin B (H&E) staining (Figure 4B) shows that cells in AL‐CMC MCs and AL GBs formed both, loosely aggregated, as well as more tightly packed spheroids. Interestingly, the spheroids in AL‐CMC1 MCs mostly form tight clusters, while the spheroids in AL‐CMC2 MCs are loosely connected (Figure S12, Supporting Information). To understand the structures of spheroids more precisely, we implemented immunostaining.

Cytoskeleton is a major determinant of cell mechanics and F‐actin is reported to drive cell migration.^[^ ^42^, ^43^, ^44^ ^]^ Keratin 8 is a cytoskeletal intermediate filament protein expressed in hepatocytes.^[^ ^45^ ^]^ An increase in F‐actin and a decrease in keratin content result in softer and more deformable tissues, or vice versa, and therefore the interplay of F‐actin and keratin enables adaptation to the mechanical demands in various tissues, e.g., such as those in lung and intestine.^[^ ^42^ ^]^ **Figure** **5** summarizes the analysis of the mechanical adaptation of the HepG2 spheroids (after 7 d of culture) to the environment with increasing viscosity within the liquid core of the MCs. Panel A in Figure 5 illustrates the differences in the volume and F‐actin and keratin 8 content, between alginate GBs and MCs. The colors in the sketch depict respective biomarkers at the membrane surface. In the AL GBs, the available alginate matrix supports the growth of spheroids (Figure 5B; Figures S13 and S14, Supporting Information). The core of AL MCs does not offer any mechanical support for cells. The lack of mechanical support leads to the production of more keratin 8 protein, which endows the cells with a more rigid cytoskeleton in liquid conditions (Figure 5B,C; Figures S15 and S16, Supporting Information). Some previous studies also demonstrated that the increased viscosity of the cell culture medium causes an upregulation of keratin 8 expression, which can uphold cell morphology and protect the cells under stress conditions.^[^ ^42^, ^45^ ^]^ Furthermore, keratin 8 can regulate the organization of actin fibers through RhoA‐ROCK signaling.^[^ ^45^, ^46^, ^47^ ^]^ In our study, the rigid keratin 8‐rich cell clusters form initial “nuclei” for further growth of the spheroids. Some of these “nuclei” succeed in growing into large spheroids with more “softer” F‐actin‐containing “shells” (see color distribution in Figure 5A,B; Figure S15, Supporting Information). Some spheroids exhibited adaptive behavior by forming protrusions to extend beyond their original boundaries in order to ensure survival. In MCs with incorporated CMC in the core, the amount of F‐actin progressively increases and pseudopodia form with the increase in viscosity of the environment, suggesting that cells efficiently probe the surrounding medium (see yellow arrows in F‐actin images, Figure 5B). This phenomenon was also showcased in a recent report, highlighting ARP2/3‐mediated actin branching as a direct cellular response to heightened viscosity.^[^ ^5^ ^]^ The findings suggest a pathway in which viscosity triggers NHE1‐dependent cell swelling, subsequently activating TRPV4‐dependent cell contractility.^[^ ^5^ ^]^ TRPV4‐mediated effects stimulate RhoA–ROCK–myosin II contractility, enabling cells to sense and adapt to increased fluid viscosity.^[^ ^5^ ^]^ Statistical analysis of multiple spheroids (*n* = 30) suggests that the increase in MC core viscosity leads to a tendency of cell “softening”, which is supported by higher detected content of F‐actin (Figure 5D). There is a certain deviation from this trend for AL‐CMC2.5 MCs, in which some spheroids grew larger and more rigid (higher keratin 8 content than in AL‐CMC1&2). Although this discrepancy is not completely clarified, the literature suggests the confinement within different concentrations of CMC polymer solutions with distinct characteristics and phase transitions as a possible cause.^[^ ^41^, ^45^ ^]^ At the microscale, CMC solution can demonstrate localized heterogeneity of physical properties, where certain regions of the solution exhibit behavior similar to elastic solids rather than viscous fluids.

Finally, the analysis of spheroids grown within GBs shows relatively high content of F‐actin and practically no keratin 8 (Figure 5B,C, black data points), resulting in a 6‐fold increase of the F‐actin/keratin 8 ratio. Dramatic reduction in keratin 8 content suggests that the stiffness of cancer cells, which was crucial to survive inside the liquid MC core environment, is no longer needed in hydrogel beads. These results unveil the great potential and ability of cancer assemblies to dynamically refine their mechanical properties in response to a specific microenvironment.

Previous studies mainly focused on the behavior of individual cells exposed to the environment with different viscous or elastic properties. For example, Khorshid tested the effect of medium viscosity on cell morphology change in different micrometer‐sized grooves and suggested that a high viscosity decreased the length and reduced mobility of endothelial cells B10D(2).^[^ ^4^ ^]^ However, Gonzalez‐Molina et al. found that the viscosity increase can enhance the migration and substrate mechanosensing of liver cancer cells.^[^ ^1^ ^]^ A recent study by Bera et al. demonstrated that elevated viscosity increases the motility of various cell types on 2D surfaces, and enhances cell dissemination from 3D tumor spheroids.^[^ ^5^ ^]^ In our 3D bioreactors, we emphasize that the adaptive nature of cells is scalable and demonstrate it with HepG2 microscopic spheroids (of ≈200 µm diameter) that finely tune their assembly and cytoskeletal structure upon interaction with the modified extracellular environment. While our focus is primarily on the assemblies of human hepatoma cancer cells, this research holds promise for advancing our understanding of the mechanical characteristics of cancer entities and their ability to adapt to diverse 3D microenvironments. These aspects will be addressed in further studies, that will also include more complex systems such as multicellular spheroid coculture models.

## 3 Conclusion

This study demonstrates significant differences in the generation, proliferation, and cytoskeleton structure of HepG2 spheroids in the absence of matrix support and influenced by changes in medium viscosity. Through an exploration of HepG2 cell adaptability within various 3D micro‐reservoirs (AL MCs, AL‐CMC MCs with different core viscosities, and GBs) fabricated using a high‐throughput microfluidic platform, it was confirmed that cell spheroids formed within hydrogel capsules were both larger and stiffer compared to those generated in fully gelled matrix due to the absence of solidified support. Immunostaining results validated that the HepG2 cells within MCs exhibited increased production of keratin 8, crucial for maintaining cell morphology and spheroid rigidity, along with heightened levels of F‐actin, facilitating cell spreading and aggregation. Several confounding factors, such as spatial variations of shell thickness, structural shell defects, nonuniform cell distribution within the MCs, and variability of individual cells, may also influence cell proliferation and aggregation behaviors. For instance, cells located near the shell may have easier access to nutrients compared to those residing in the MC center, which promotes faster proliferation. Our findings underscore the adaptability of cells to varying microenvironments and reveal distinct aggregation patterns in reactors with different properties.

Obtained results encourage us to perform a deeper investigation into cellular responses to more advanced mechanical cues, such as engineered spatially varying matrix stiffness or hydraulic pressure, as well as cell behaviors within more complex biologically relevant systems, such as cocultures of liver cancer cells with adipocytes.

Looking ahead, understanding the adaptation of cells to their immediate extracellular environment is crucial for addressing the most critical aspect of cancer – metastatic progression. Spheroid models can effectively represent different stages of tumor development and offer immense potential in the fields of biology and medicine. The ability to manipulate various parameters in the process paves the way for a better understanding of cancer. In this context, the cultivation and engineering of complex multicellular spheroids and organoids are particularly promising.

## 4 Experimental Section

### Materials

In this study, the following reagents were used: sodium alginate (NaAL, Sigma‐Aldrich, 120 000–190 000 g mol^−1^, M/G = 1.56), sodium carboxymethylcellulose (CMC, Sigma‐Aldrich, low viscosity, degree of substitution: 0.65–0.90), sodium dodecyl sulfate (SDS, Sigma‐Aldrich, 98.5%), CaCl_2_ (Merck, 99.5%), hexane (Fisher Scientific, 95%), sodium fluorescein (FSC, Sigma‐Aldrich, 376.27 Da), acetic acid (Carl Roth, 100%), 0.5% trypsin‐EDTA (Gibco), sucrose (Carl Roth), gelatin (Carl Roth), calcein AM (VWR (Corning)), propidium iodide (PI) (VWR (Corning)), hematoxylin (Sigma‐Aldrich), eosin B (Sigma‐Aldrich), RotiMount (Carl Roth), Roticlear (Carl Roth). All reagents were used as received without additional purification.

### Viscosity of Cell Culture Medium with or without CMC

Both the viscosity and the density of Dulbecco's Modified Eagle Medium (DMEM, + 4.5 g L^−1^ d‐Glucose, – sodium pyruvate, Gibco) with 0, 1, 2, and 3% w/v of added CMC were determined with a viscometer (Kinematic viscometer SVM 3001, Anton Paar) at cell incubator temperature (37 °C) and room temperature (20 °C). Each sample was drawn up from the storage vessel using a syringe. The sample was degassed to prevent bubble formation during the measurement by creating a negative pressure in the syringe.

### Preparation of AL MCs, AL‐CMC MCs, and AL GBs

AL and AL‐CMC MCs were prepared at room temperature (20 °C) using our in‐house built cross junction‐ and PTFE tubing‐based microfluidic platform, as described previously.^[^ ^29^ ^]^ Briefly, cell culture medium with 0, 1, or 2% w/v of CMC, 3% w/v NaAL with 0.5 × 10^−3^ m SDS, and hexane were used as inner fluid (IF), middle fluid (MF), and outer fluid (OF), respectively. The flow rates were: *q*_IF_ = 1 mL h^−1^, *q*_MF_ = 1.5 mL h^−1^, and *q*_OF_ = 20 mL h^−1^. A continuous co‐flow of NaAL and cell culture medium was formed with the assistance of SDS at the first cross‐junction. When the co‐flow reached the second cross‐junction, a stream of aqueous droplets was formed. A 200 × 10^−3^ m CaCl_2_ gelation bath (CB) was used to collect the droplets and convert them into capsules within 5 min.

To prepare AL GBs, only one cross junction was employed. 1.5% w/v NaAL solution in cell culture medium was used as IF. Hexane was used as OF. The flow rates were: *q*_IF_ = 2.5 mL h^−1^ and *q*_OF_ = 20 mL h^−1^.

After transferring the MCs and GBs into the cell culture medium, the diameters and shell thicknesses of MCs and GBs were manually measured using ImageJ (*n* = 50).

### Permeation Experiments

To test the differences in diffusion rate during the loading of molecules into AL MCs, AL‐CMC MCs, and AL GBs, permeation experiments were carried out with a 5 × 10^−3^ m solution of sodium fluorescein (FSC, *M*_r_ = 376 Da) in the cell culture medium. The images were acquired at 1 s intervals (*n* ≥ 3) with a 10× objective in an upright laser‐scanning confocal fluorescence microscope (Olympus IX83).

### Cell Culture

HepG2 Red Fluc (referred to as HepG2 in this study) cells were cultured in Dulbecco's Modified Eagle Medium (DMEM, + 4.5 g L^−1^ d‐Glucose, – Sodium Pyruvate, Gibco) supplemented with 1% streptomycin and penicillin (Biochrom), and 10% fetal bovine serum (Biochrom) in a cell incubator (5% CO_2_ at 37 °C). Cells were utilized in passage ranges P16‐25.

### Cell Proliferation and Viability Tests for Different Concentrations of Incorporated CMC in 2D Plate Culture

To test cell proliferation in a 2D environment and assess the influence of CMC concentration on cell viability, cell culture media with 0, 1, 2, and 3% w/v of CMC were used to culture HepG2 cells with a density of 1×10^6^ cell mL^−1^ using 6‐well plates (2 mL of cell culture medium per well) in a cell incubator (5% CO_2_ at 37 °C). After 2 d of culturing, cells were observed, and images were recorded using a microscope (ECLIPSE TS100, Nikon).

For the cell viability assay, cells were then trypsinized and the viability was tested using calcein AM/PI (live/dead) assay adapted according to the protocol from the supplier (VWR (Corning)). First, 16 µL of calcein AM solution (400 µg mL^−1^) was added to each well. After 45 min, 8 µL of PI solution (500 µg mL^−1^) was added to further incubate for 1 min. An optical microscope (Axio Observer, Zeiss) with a green‐light detection mode (filter set: 495 nm beamsplitter, 450–490 nm excitation, and 500–550 nm emission) and a red‐light detection mode (filter set: 570 nm beamsplitter, 538–562 nm excitation, and 570–640 nm emission) was used to acquire the images. NIVA software^[^ ^48^ ^]^ was used to obtain and process viability data.

### Cell Encapsulation

To prepare AL and AL‐CMC MCs with HepG2 cells, the cell culture medium (cell density = 1–1.2×10^7^ cell mL^−1^) with 0, 1, 2, or 2.5% w/v of CMC was used as inner fluid (IF).

To prepare AL GBs with HepG2 cells, 1 mL of cell culture medium with a cell density of 2–2.4×10^7^ cell mL^−1^ was mixed with 1 mL of 3% w/v NaAL to obtain the NaAL solution in cell culture medium with a final cell density of 1–1.2×10^7^ cell mL^−1^. This solution was used as IF.

The generated cell‐laden MCs and GBs were washed once with cell culture medium and finally cultured in the 24‐well plate (1 mL of cell culture medium per well) in the cell incubator. The culturing medium was replaced with a fresh medium every 2–3 d.

### Cell Density Counting and Cell Viability in MCs and GBs

Cell culture medium at different culturing periods of MCs and GBs was exchanged with a mixed solution (of volume *V*) containing 450 µL of 55 × 10^−3^ m sodium citrate and 100 µL of trypsin (0.05%). After culturing at 37 °C for 30 min, the raw cell density (*D*_raw_) and cell viabilities were measured with Casy‐ton (3×3 cycles, measuring capillary: 150 µm). Numbers of MCs and GBs (*X*_MCs/GBs_) with cell spheroids were counted under the microscope. The average volume of each MC core containing cells was defined as *V*_MCcore_ = 0.0335 µL. This volume was calculated by measuring the core diameter of *n* ≥ 50 MCs that were assumed to have spherical geometry. The average volume of each GB containing cells was defined as *V*_GB_ = 0.1796 µL (calculated by measuring the diameter of *n* ≥ 50 GBs). The final average cell density in MCs or GBs was calculated as

(1) $$\mathrm{Cell}\;\mathrm{density}\;\mathrm{in}\;\mathrm{MC}/\mathrm{GB}=\frac{V\;\times \;D_{\mathrm{raw}}}{X_{\mathrm{MCs}/\mathrm{GBs}}\;\times \;V_{\mathrm{MCcore}/\mathrm{GB}}}$$

### Spheroid Slides and H&E Staining

H&E staining was conducted according to the previously reported protocol.^[^ ^29^ ^]^ Briefly, HepG2 spheroids in AL MCs, AL‐CMC MCs, and AL GBs were prepared in advance in a 24‐well plate and cultured at 37 °C under 5% CO_2_ for 7 d. MCs or GBs were then embedded into a mixed gel which contained 20% of sucrose and 7.5% of gelatin. A microtome cryostat (CM1850, Leica) was used to cut the samples into 20 µm thick films to produce slides. The nucleus and cytoplasm were stained using hematoxylin and eosin B for 3 min and 30 s, respectively. Images were captured using an Axio Imager A1 Microscope (Zeiss).

### Spheroid Live/Dead Staining and Morphology

To record the proliferation of cells in MCs and GBs, bright field images and live/dead staining images were obtained. The staining method and imaging process were the same as described in 4.6. The numbers of spheroids in MCs and GBs (*n* ≥ 10) were counted. The morphology of spheroids in either MCs or GBs was analyzed using ImageJ by manually drawing the boundary and then measuring the Feret diameter. The minimum volumes of spheroids were chosen based on spheroid proliferation within each group (see Table S4 in the Supporting Information for details). The volume cut‐offs were selected to eliminate 1/3 of the spheroids with the smallest size and the largest size deviations.

To quantify the increase in size and morphology of the spheroids in MCs and GBs, we approximated the spheroids with ellipsoids (see inset of Figure S5 in the Supporting Information). The volume of the spheroids (*V*) was estimated using the following equation

(2) $$V=\frac{1}{6}\pi abc$$

where *a* is the long radius, *b* and *c* are the short radii (considered to be equal), and ***π*** is the mathematical constant.

Circularities of spheroids were analyzed by calculating the sphericity factor (SF) according to the previous report^[^ ^49^ ^]^

(3) $$\mathrm{SF}\;=\frac{a-b}{a+b}$$

When the value of SF is 0, the shape of the spheroid is considered as perfectly spherical.

### Immunostaining of Spheroids in MCs and GBs

To visualize the 3D structure and development of spheroids, F‐actin, keratin 8, and nuclei were stained with phalloidin (conjugated with Alexa Fluor 488, ThermoFisher, 1:200), cytokeratin 8 (conjugated with Alexa Fluor 647, Covance, 1:100), and Hoechst 33 258 (ThermoFisher, 1:10), respectively. Precisely, spheroids in capsules were precultured for 7 d, transferred to 18 µ‐slides, and washed three times with PBS. Afterward, PBS containing 4% paraformaldehyde was used to fix the spheroids for 30 min. 10 × 10^−3^ m EDTA was then used to incubate the capsules for 5 min, dissolve them, and release the spheroids. After permeabilization with 0.2% Triton X‐100 for 10 min, the spheroids were blocked using 3% BSA for 2 h. Primary antibody (cytokeratin 8) was then added to incubate the spheroids overnight at 4 °C. After washing three times with the blocking solution, secondary antibodies phalloidin and Hoechst 33 258 were added for 30 and 10 min incubation at room temperature, respectively. Finally, Prolong (ThermoFisher) antifade reagent was added to prevent the quenching of fluorescence. The stained spheroids were imaged using a confocal fluorescence microscope (Olympus IX83).

### F‐actin and Cytokeratin 8 Ratio

To measure F‐actin and cytokeratin 8 staining ratio, fluorescent micrographs of F‐actin and cytokeratin 8 were acquired after immunostaining with a 20× or a 40× objective in an upright laser‐scanning confocal fluorescence microscope (Olympus IX83). The laser intensity and exposure time were kept constant during the acquisition. The average intensity and area of F‐actin and cytokeratin 8 staining per spheroid were quantified using ImageJ. Finally, the ratios of fluorescent area and mean intensity were calculated.

### Statistical Analysis

Data are reported as mean ± standard deviation (SD). p‐values were calculated using the two‐way ANOVA test combined with Tukey's or Dunnett's multiple comparison test using GraphPad Prism 9 depending on the specific experiment. Differences between experimental groups were considered significant when *p < 0.0332, **p < 0.0021, ***p < 0.0002, and ****p < 0.0001, *n* ≥ 3.

## Conflict of Interest

The authors declare no conflict of interest.

## Supporting information

Supporting Information

Supplemental Video 1

Supplemental Video 2

## Data Availability

The data that support the findings of this study are available in the supplementary material of this article.
