# Supplementary material for: Impact of Viscosity on Human Hepatoma Spheroids in Soft Core–Shell Microcapsules
Source: Adv Healthc Mater. 2024 Jan 25;13(11):2302609. doi: 10.1002/adhm.202302609 (PMC11468952; doi:10.1002/adhm.202302609)
Supplement: Supplementary file 1 — Supporting Information [file ADHM-13-2302609-s001.pdf]

ADVANCED HEALTHCARE MATERIALS

Supporting Information

for *Adv. Healthcare Mater.*, DOI 10.1002/adhm.202302609

Impact of Viscosity on Human Hepatoma Spheroids in Soft Core–Shell Microcapsules

*Xuan Peng, Željko Janićijević, Sandy Lemm, Sandra Hauser, Michael Knobel, Jens Pietzsch, Michael Bachmann and Larysa Baraban**

Impact of Viscosity on Human Hepatoma Spheroids in Soft Core-Shell Microcapsules

Xuan Peng, Željko Janićijević, Sandy Lemm, Sandra Hauser, Michael Knobel, Jens Pietzsch, Michael Bachmann, Larysa Baraban

Table S1. List of terms

Terms	Description
Liver cancer	Liver cancer is a cancer that starts in the liver. Liver cancer can be primary (starts in the liver) or secondary (cancer from elsewhere spread to the liver).[1]
Hepatocellular carcinoma	Hepatocellular carcinoma is also called hepatoma or HCC, which is the most common type of liver cancer (accounts for ~90% of cases).[2, 3]
HepG2	HepG2 is a human hepatoma cell line, which is considered as a suitable model for hepatocellular carcinoma.[4, 5]
Microtissue	There is no clear and established definition of microtissue so far;[6] Here, we refer to microscale aggregations of seeded cells (diameter of spheroids < 1000 µm).

Table S2. Summary of recent research regarding cell response to different extracellular physical cues.

Physical cues	Cell culture	Scope/Main conclusion	Reference
**Viscosity**	Human MDA-MB-231 cell lines (breast), HOS (bone), U87 (brain), HEK293 (kidney), and dermal fibroblasts.	Viscosity increases the motility of various cell types on 2D surfaces and increases cell dissemination from 3D tumor spheroids.	K. Bera, et al. **Nature** 2022.
	HepG2, SKHEP1, and Huh7. (liver)	Fluid viscosity enhances liver cancer cell mechanosensing and migration, studied on 2D plate of different surfaces.	J. Gonzalez-Molina, et al. **Biomaterials** 2018.
	Epitenon cells, and mouse endothelial	Viscosity of the medium alter the cellular	F. A. Khorshid, et

	B10D(2) cells.	reaction to topography, studied on 2D surface.	al. **Proc. 2nd Saudi Sci. Conl, Fac. Sci.** 2005.
**Viscoelasticity**	MCF10A (breast), and intestinal organoids.	Viscoelasticity controls spatiotemporal tissue organization, studied using alginate hydrogel and computational modelling.	A. Elosegui-Artola, et al. **Nat. Mater.** 2023.
	Human dermal fibroblasts, ATDC5 chondrocyte, Human mesenchymal stem cells.	Modular mixing hydrogelators control cell aggregation in 3D, studied using benzene-1,3,5-tricarboxamide based fibrous hydrogel.	S. Hafeez et al. **Biomater. Sci.** 2022.
**Elasticity / Stiffness**	L929 (fibroblast), HeLa (cervical cancer), and MCFs (breast)	Cell spreading is suppressed at an increasing stress-relaxation amplitude with a fixed elasticity and stress-relaxation timescale, studied using a hybrid hydrogel,	W. Yu, et al., **Int. J. Mol. Sci.**, 2022.
	Human neutrophils	Neutrophil adhesion increases with substrate stiffness, leading to different neutrophil extracellular traps under different potent inducers stimulate, studied on 2D surface.	L. Erpenbeck, et al. **Front. Immunol.** 2019.
	Human myeloid leukemia cell lines	Stiffness causes variations in proliferation and chemosensitivity in myeloid leukemias, studied in alginate hydrogels.	J.-W. Shina, et al., **PNAS**, 2016.
	Multipotent subpopulations of c-kitþ progenitor cells	Elasticity controls expansion, morphology, gene expression, and cell migration, studied on 2D surface.	A. Skardal, et al. **J. Mech. Behav. Biomed. Mater.** 2013.

	Human bone marrow stem cells	The combined effect of surface topography and substrate rigidity on cell morphology was studied on 2D surface.	S. Ribeiro, et al. **Eng. Life Sci.** 2022.
**Topography**	Human corneal stromal cells	Different cellular polarization, adhesion, gene expression, and migration were studied on 2D surface.	P. Bhattacharjee, et al. **Colloids Surf. B** 2020
	MCF-10A (non-cancer human breast epithelial cells), MDA-MB-231, MCF-7 (human malignant breast cancer cell line)	Topographic cues could activate Rho-ROCK-Myosin signaling to suppress non-cancerous cell proliferation. Studied on 2D surface.	P. K. Chaudhuri, et al. **Sci. Rep.** 2016.

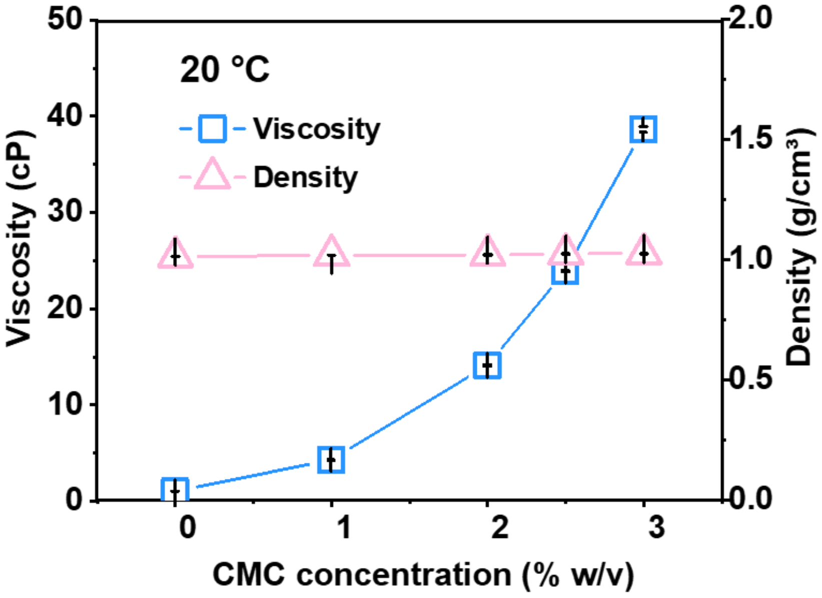

Figure S1. Viscosity and density of DMEM cell culture medium for a series of concentrations of added CMC (0, 1, 2, 2.5, 3% w/v) measured at 20 °C.

Table S3. Viscosity and density of DMEM cell culture medium for a series of concentrations of added CMC.

	37 ℃		20 ℃	
CMC (wt%)	Viscosity (cP)	Density (g/cm^3)	Viscosity (cP)	Density (g/cm^3)
0	0.8185±0.0055	1.0006±0.0008	1.0201±0.0021	1.0114±0.0012
1	2.6944±0.0120	1.0072±0.0001	4.2928±0.0142	1.0172±0.0000
2	8.2276±0.0061	1.0109±0.0001	14.107±0.0961	1.0192±0.0015
2.5	13.4987±0.1085	1.0337±0.0266	23.915±0.0924	1.023±0.0011
3	21.8800±0.1478	1.0159±0.0003	38.6507±0.3216	1.0242±0.0015

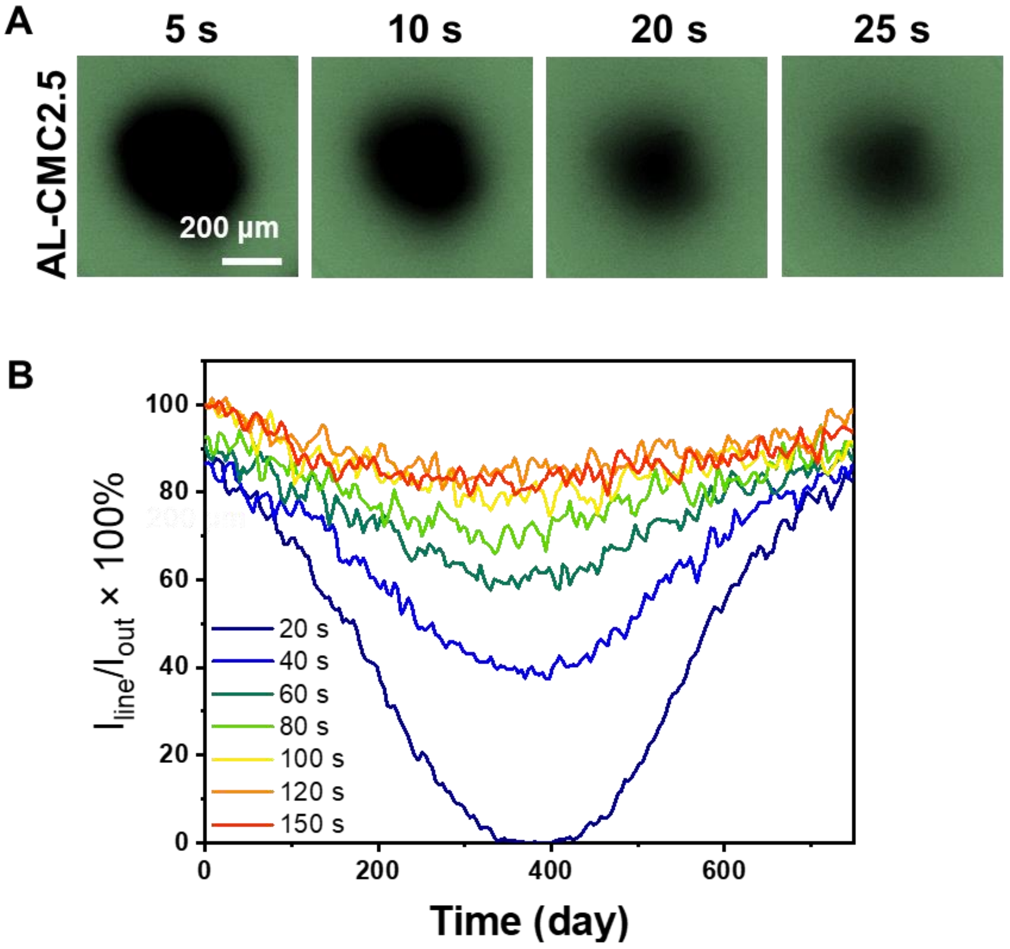

Figure S2. (A) Representative fluorescent micrographs of AL-CMC2.5 MCs obtained after immersion in 5 mM solution of sodium fluorescein (FSC) (376 Da) and (B) Midline/outer fluorescence intensity ratio for AL-CMC2.5 MCs at different time points.

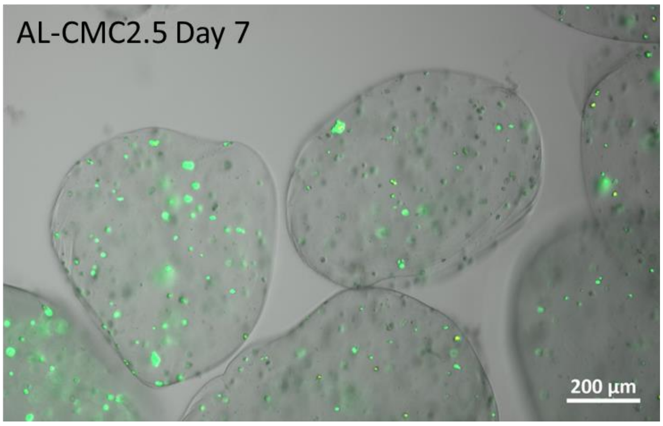

Figure S3. Representative image of cell proliferation in AL-CMC2.5 MCs on day 7.

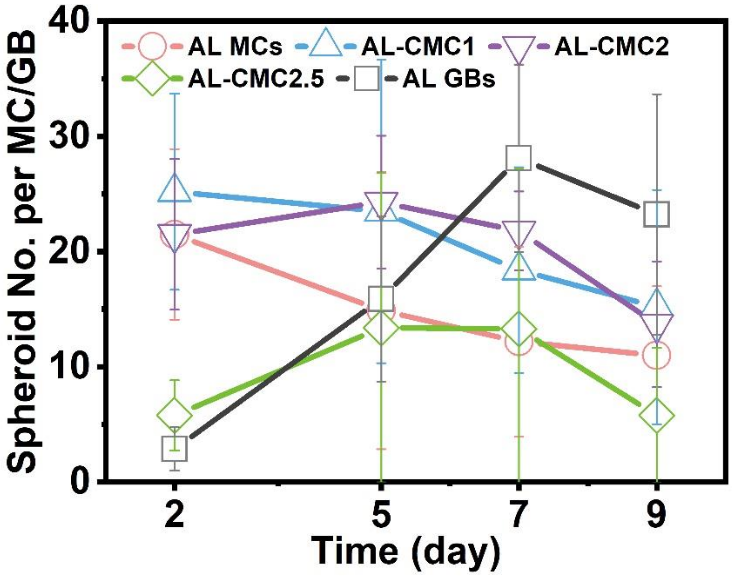

Figure S4. Average number of spheroids per MC & GB (n≥10).

Table S4. Minimum volume of individual spheroids which was accounted for in the size comparison between spheroids generated in MCs or GBs. Only the individual spheroids with larger or equal volume were considered in the calculations.

V_{min} (× 10^5 μm^3)	Day 2	Day 5	Day 7	Day 9
AL MCs	0.02	2	2	4
AL-CMC MCs	0.02	0.06	0.15	0.2
AL GBs	0.02	0.06	0.06	0.1

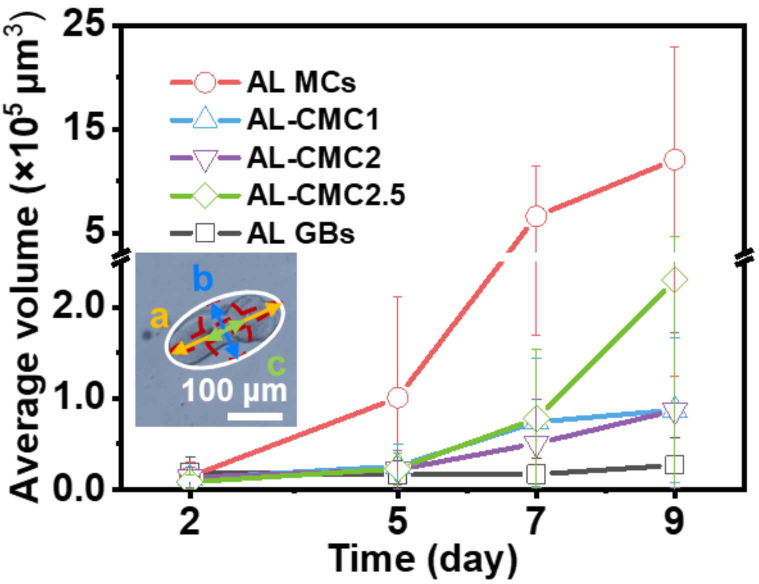

Figure S5. Spheroid volumes obtained in MCs with 0, 1, 2 and 2.5% w/v of CMC in the core and in AL GBs. (n≥13)

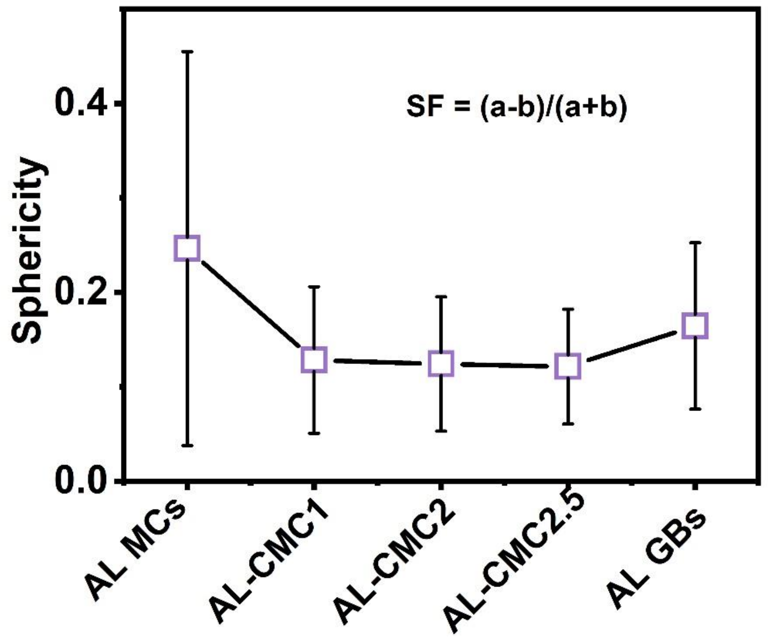

Figure S6. Circularity of spheroids in MCs and GBs. (n ≥ 100) The sphericity factor (SF) was calculated according to $SF = \frac{a-b}{a+b}$, where a is the long radius, and b is the short radii.

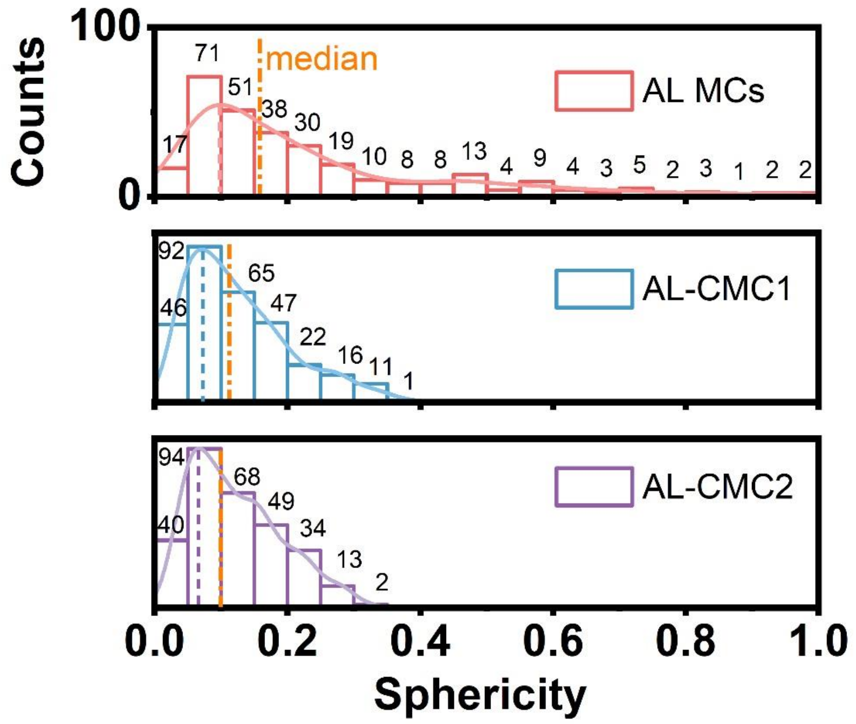

Figure S7. Circularity distribution of spheroids in AL, AL-CMC1, and AL-CMC2 MCs. (n = 300) The distributions were fitted using Kernel Smooth fitting. The dashed lines represent the peaks of the fitting curves. The dash-dotted lines designate the

median values of sphericity.

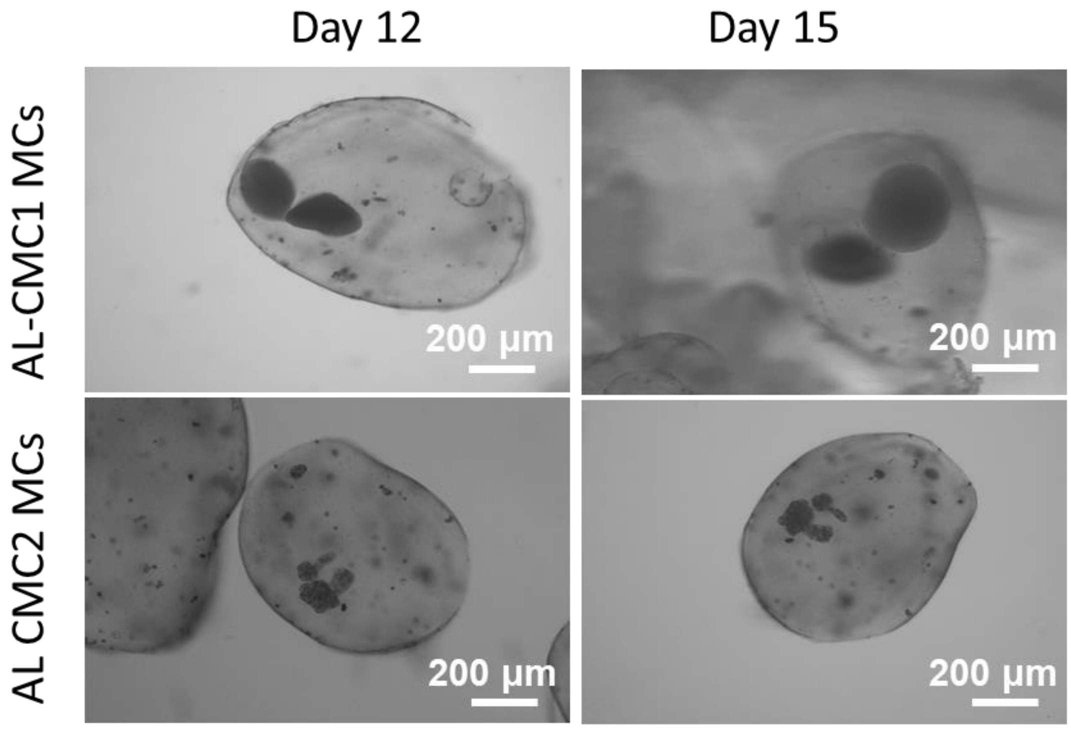

Figure S8. Representative images of cell proliferation in AL-CMC MCs on day 12 and day 15.

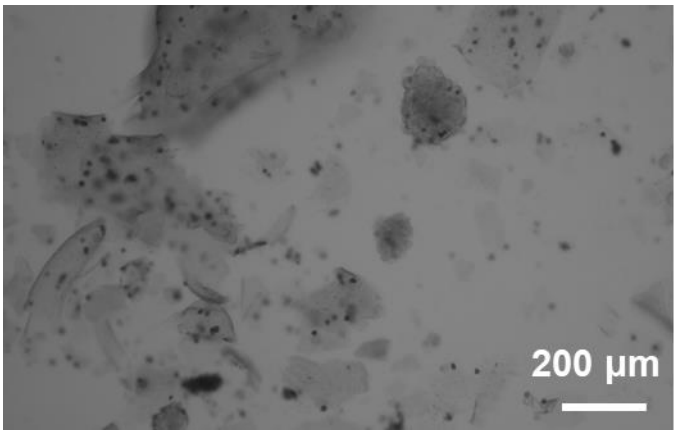

Figure S9. Representative images of alginate fragments obtained after 9 days of culturing.

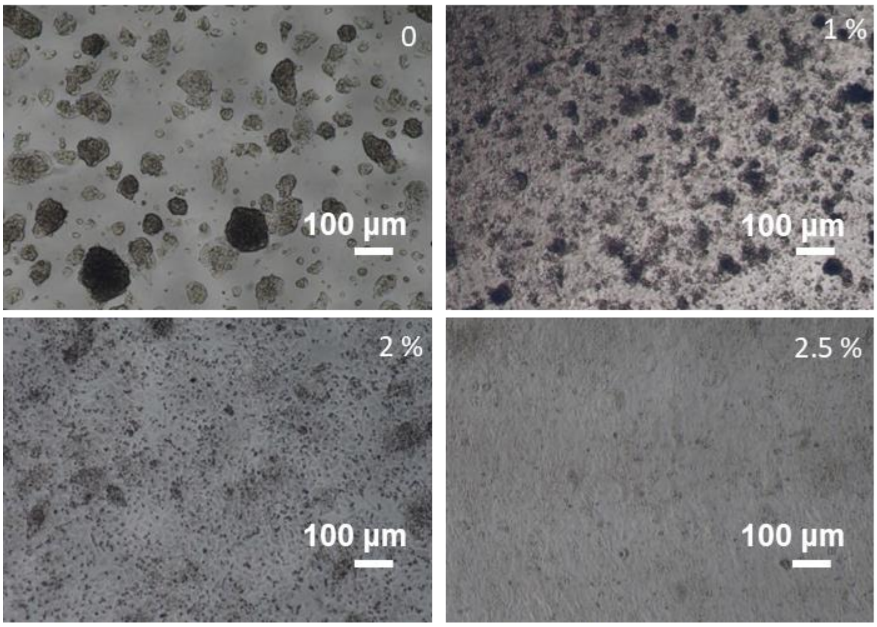

Figure S10. Cell proliferation on a 6-well plate during culturing in the medium with 0, 1, 2, and 2.5% w/v of CMC.

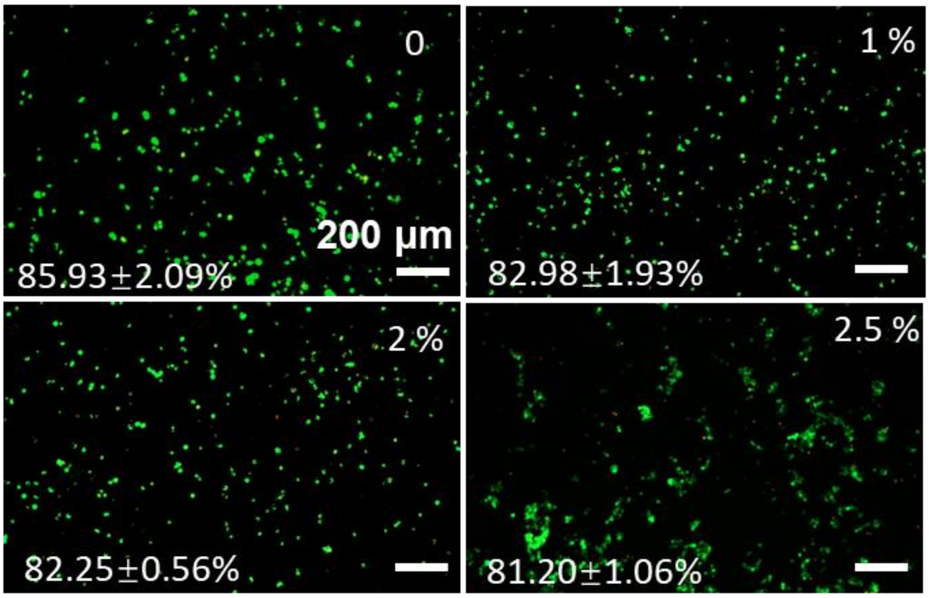

Figure S11. Cell viability on day 2 after culturing on a plate in the medium with 0, 1, 2, and 2.5% w/v of CMC.

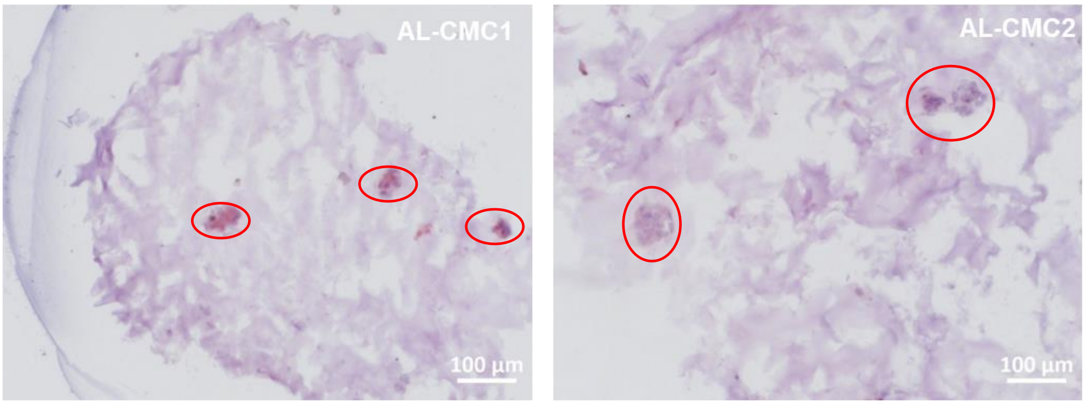

Figure S12. H&E staining of cell clusters in AL-CMC1 and AL-CMC2 MCs.

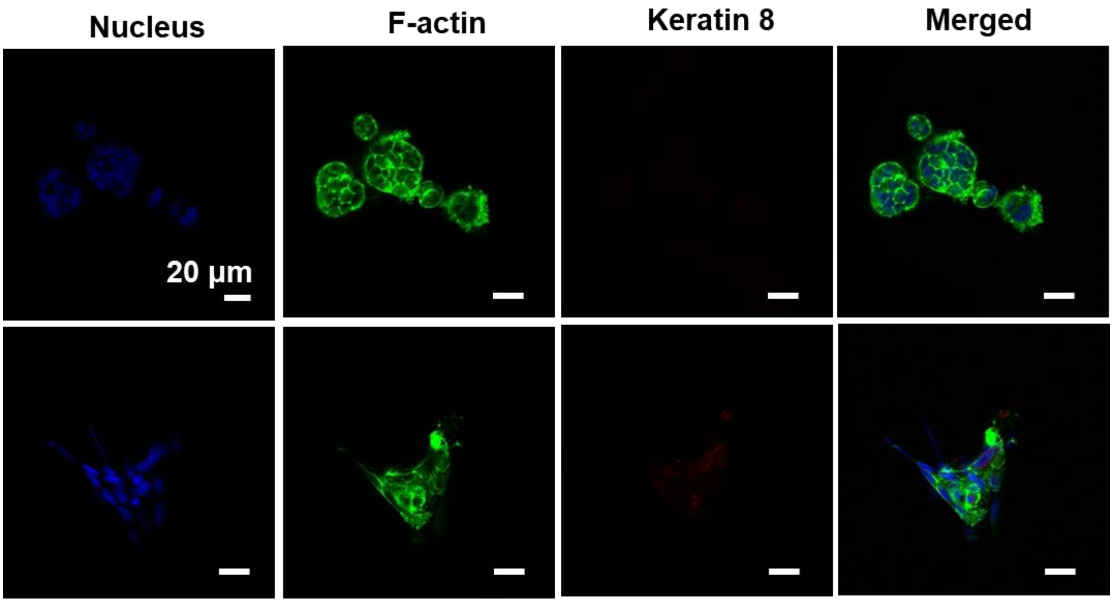

Figure S13. Representative immunostaining images of HepG2 spheroids in AL GBs.

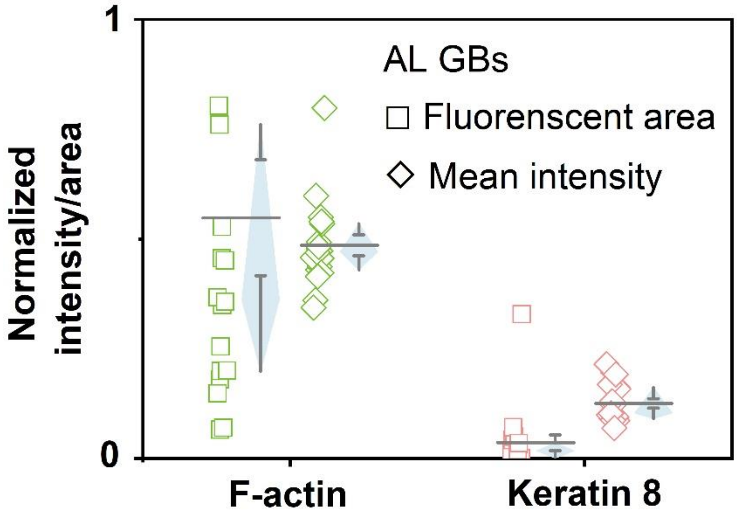

Figure S14. Box plot of normalized intensities of F-actin and keratin 8 measured from fluorescent micrographs of spheroids formed in AL GBs (n = 18).

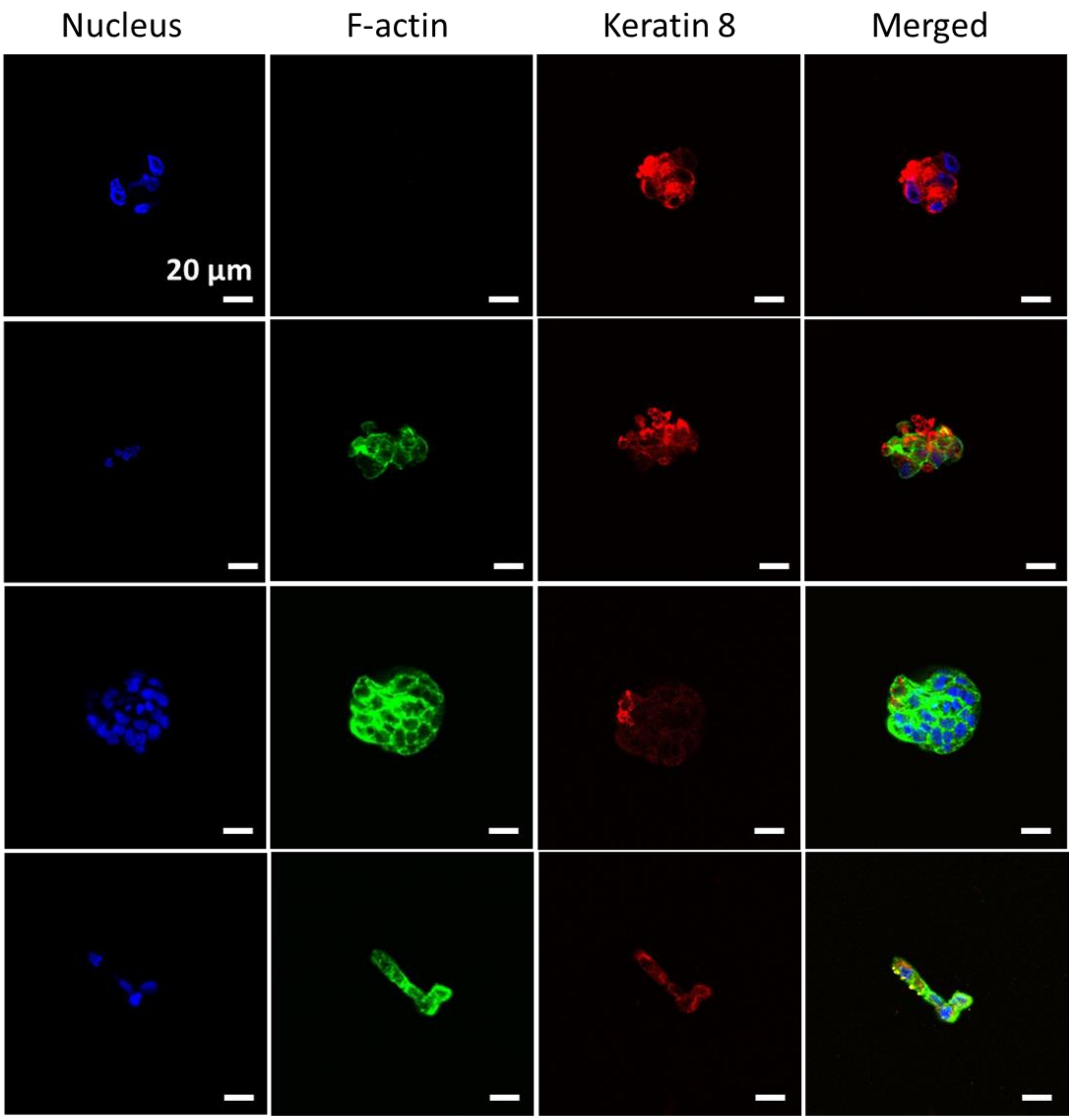

Figure S15. Representative immunostaining images of spheroids in AL MCs.

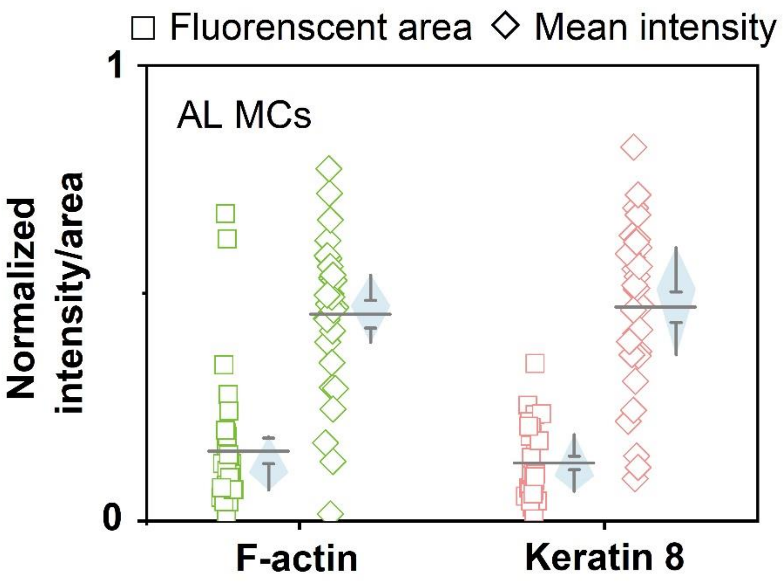

Figure S16. Box plot of normalized intensities of F-actin and keratin 8 measured from fluorescent micrographs of spheroids formed in AL MCs (n = 30).

[1] C. R. UK, What is liver cancer?, https://www.cancerresearchuk.org/about-cancer/liver-cancer/about-liver-cancer, accessed: **Jun., 2021**.

[2] J. M. Llovet, R. K. Kelley, A. Villanueva, A. G. Singal, E. Pikarsky, S. Roayaie, R. Lencioni, K. Koike, J. Zucman-Rossi, R. S. Finn, *Nat. Rev. Dis. Primers* **2021**, *7*, 6.

[3] C. R. UK, Types of liver cancer, https://www.cancerresearchuk.org/about-cancer/liver-cancer/types, accessed: **Sep., 2021**.

[4] S. Lemm, S. Kohler, R. Wodtke, F. Jung, J. H. Kupper, J. Pietzsch, M. Laube, *Cells* **2022**, *11*.

[5] A. Filliol, R. F. Schwabe, *Nat. Metab.* **2022**, *4*, 1225.

[6] J. Zhang, W. Xu, C. Li, F. Meng, Y. Guan, X. Liu, J. Zhao, J. Peng, Y. Wang, *Tissue Eng. Part B Rev.* **2021**, *28*, 393.
